# Molecular Characterization of *Anopheles algeriensis* Theobald, 1903 (Diptera: Culicidae) Populations from Europe

**DOI:** 10.3390/pathogens11090990

**Published:** 2022-08-30

**Authors:** Michela Menegon, Alexandru Tomazatos, Francesco Severini, Donato Antonio Raele, Tobias Lilja, Doreen Werner, Daniela Boccolini, Luciano Toma, Ilaria Vasco, Renke Lühken, Helge Kampen, Maria Assunta Cafiero, Marco Di Luca

**Affiliations:** 1 Dipartimento Malattie Infettive, Reparto Malattie Trasmesse da Vettori, Istituto Superiore di Sanità, 00161 Rome, Italy; 2 Department of Arbovirology, Bernhard Nocht Institute for Tropical Medicine, 20359 Hamburg, Germany; 3 Laboratorio di Entomologia Sanitaria, Istituto Zooprofilattico Sperimentale della Puglia e della Basilicata, 71121 Foggia, Italy; 4 Department of Microbiology, National Veterinary Institute, 75189 Uppsala, Sweden; 5 Leibniz Centre for Agricultural Landscape Research (ZALF), 15374 Müncheberg, Germany; 6 Friedrich-Loeffler-Institut, Federal Research Institute for Animal Health, 17493 Greifswald, Germany

**Keywords:** *Anopheles algeriensis*, barcoding, mitochondrial cytochrome c oxidase subunit I, intergenic transcribed spacer 2, phylogenetic analysis, cryptic species

## Abstract

*Anopheles algeriensis* Theobald, 1903, considered a competent vector of *Plasmodium* parasites, is a mosquito species widely distributed in the Mediterranean area but rare in Northern and Central Europe. The disappearance of its suitable breeding sites in Italy is having a detrimental effect on the occurrence of this species once common along the Southern coasts and on the islands. Recently, molecular investigations have renewed interest in this species, highlighting a genetic heterogeneity among European populations. In this study, *An. algeriensis* populations from Italy, Germany, Romania, and Sweden were analyzed by molecular typing of the intergenic transcribed spacer 2 (ITS2). The mitochondrial cytochrome c oxidase subunit I (COI) was also analyzed from specimens collected in Southern Italy. With the aim of investigating the population structure of this species, the obtained data were compared to all publicly available ITS2 and COI sequences of *An. algeriensis*, adding specimens from Spain and Portugal. The analyses of both markers indicate a split between Iberian populations (Spain for ITS2 and Spain/Portugal for COI) and those from the rest of Europe, revealing two cryptic species. The analysis of the COI barcode revealed a third clade representing a cryptic species present in Danube Delta (Romania). The high levels of genetic divergence among the clades of *An. algeriensis* indicate that this taxon represents a species complex, potentially harboring several distinct cryptic species.

## 1. Introduction

Mosquito-borne diseases represent a global threat to human and animal populations [1]. Among them, malaria still kills more than 500,000 people each year. In 2020, the estimated figure for malaria deaths was 627,000, representing an increase of 12% compared to the previous year due to service disruptions during the COVID-19 pandemic [2]. *Plasmodium falciparum* is the deadliest malaria parasite and the most prevalent in the WHO African Region, accounting for about 96% of deaths globally. *Plasmodium vivax* is the dominant malaria parasite in most countries outside of sub-Saharan Africa and was responsible for 2% of all globally estimated cases in 2020 [2].

Although malaria is no longer an endemic disease throughout Europe, in recent years, several locally acquired malaria cases have occurred in Greece, France, Spain, and Lithuania [3,4] along with cryptic cases in Italy [5]. These events fuel concerns that ongoing climate and demographic changes may contribute to the re-emergence of malaria in Central and Southern Europe [6]. Areas where mosquito species competent for *Plasmodium* transmission are still present in epidemiologically relevant densities are particularly vulnerable. For this reason, research activities on the biological and ecological aspects of *Anopheles* mosquitoes, including vector species of minor importance such as *Anopheles algeriensis*, can contribute to the surveillance and control of the re-emergence of malaria in Europe [7].

*Anopheles algeriensis* Theobald, 1903 is a thermophilic mosquito widely distributed in Asia and Europe. This species is clearly more common in Mediterranean countries [8,9], but its presence has been reported in several Northern European countries [10,11,12,13]. Moreover, its distribution range seems to extend to North Africa [14], including Algeria and Morocco [15,16]. This species overcomes the cold season as larvae in small water bodies that are rich in vegetation [17].

In Italy, the progressive disappearance of larval habitats has been considerably affecting the survival and presence of this species, once common along southern coasts, and in Sicily and Sardinia [18,19]. However, recent monitoring of malaria vectors in Southern Italy has found that *An. algeriensis* occurs in high densities in the Apulia and Basilicata regions [20].

Although there is no definite evidence for the vector competence of this species, *An. algeriensis* is historically considered to be a secondary vector of malaria in Europe [7]. In addition, this species was considered responsible for episodic malaria epidemics in Algeria during the early part of the 20th century [21]. The species was also deemed a secondary vector in Morocco, but its role in transmission was negligible due to its low abundance in the country [15,22].

The mitochondrial cytochrome c oxidase subunit I (COI) gene has been commonly used for DNA barcoding in animals [23], and is the most widely used molecular marker in the identification of genetic variations and phylogenetic relationships among mosquitoes [24]. In particular, a high degree of intra- and inter-population genetic diversity was observed in *An. algeriensis* specimens from Romania [10] and between populations from Spain and Germany or Sweden [25]. A second molecular marker, the intergenic transcribed spacer 2 (ITS2), is extensively used to determine phylogenetic relationships among closely related *Anopheles* species [26,27,28,29].

The aim of the present study was to investigate the genetic diversity and structure of this species over a wide geographical range in Europe. To this end, we used Bayesian phylogenetics, tree-based and distance-based approaches for exploring the genetic diversity of *An. algeriensis* populations from six European countries.

## 2. Results

**Figure 1:**
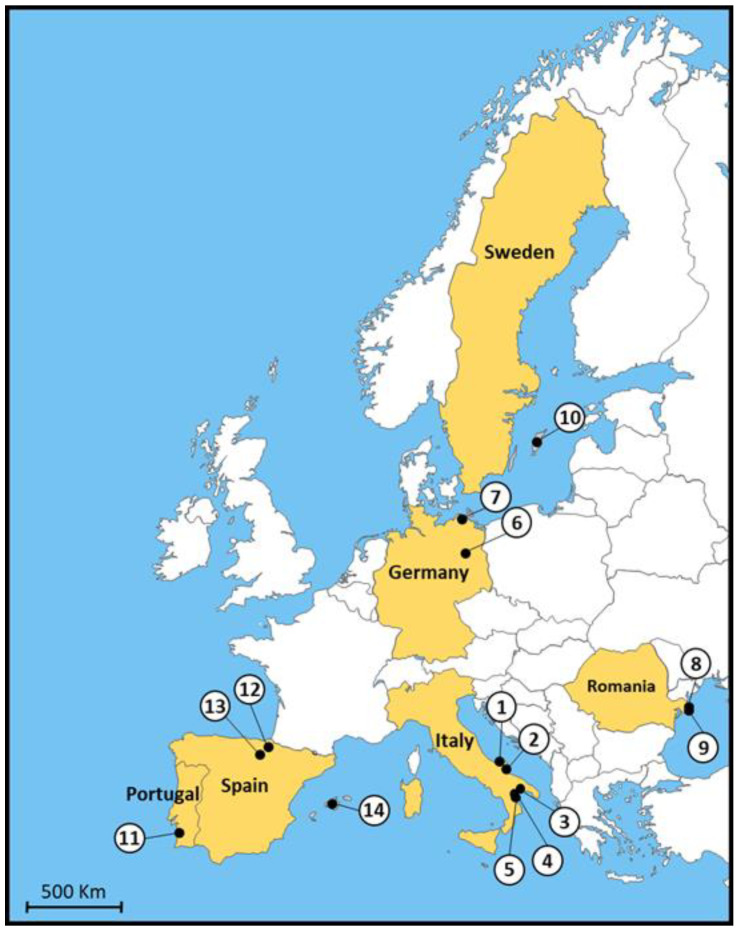
Map of sites of origin of *Anopheles algeriensis* specimens analyzed in this study. (1) Lesina (Foggia Province-Apulia); (2) Manfredonia (Foggia Province-Apulia); (3) Castellaneta (Taranto Province-Apulia); (4) Pisticci (Matera Province-Basilicata); (5) Policoro (Matera Province-Basilicata); (6) Klein Behnitz (Federal State of Brandenburg); (7) Wustrow (Federal State of Mecklenburg-Western Pomerania); (8) Sulina (Danube Delta Biosphere Reserve); (9) Lake Roşuleţ (Danube Delta Biosphere Reserve); (10) Gotland (Gotland Island); (11) Santiago do Cacém (Alenteyo Region); (12) Navarra Province; (13) Rioja Province; and (14) Majorca Island.

We obtained ITS2 sequences from 128 mosquitoes collected in Italy, Germany, Romania, and Sweden. A subset of 43 *An. algeriensis* from Southern Italy and one specimen from Romania were sequenced for a fragment of the COI gene. Details of samples sequenced for ITS2 and COI are provided in Table S1. The generated sequences were compared with homologs retrieved from Genbank (Table S2): 48 COI sequences and 13 ITS2 sequences, enabling a more comprehensive comparison of European populations of *An. algeriensis* (Figure 1).

### 2.1. Phylogenetic Analysis and Species Delimitation Based on COI Sequences

A fragment of the COI gene (692 base pairs [bp]) was successfully amplified for 42 specimens of *An. algeriensis* from Apulia and one specimen from Basilicata (Table S1).

The COI sequences obtained showed up to 18 single nucleotides polymorphisms (SNPs), identifying 16 haplotypes (Table S3) with a pairwise identity of 97.4–99.9%. The most frequent haplotype was shared by ten specimens, and the rest of the haplotypes accounted for one to four specimens.

In order to extend the molecular investigation to the *An. algeriensis* populations from other countries, all homologous COI sequences available in GenBank were analyzed (Table S2). Specifically, eigth COI sequences from Germany (709 bp), three from Portugal (658 bp), 14 from Romania (from 519 to 552 bp), 21 from Spain (from 590 to 658 bp), and two from Sweden (658 bp) (Table S2). Two additional Swedish sequences (KP942713-KP942514), which were too short to be compared with the other available COI sequences, were not considered for analysis. In addition, a further original COI sequence obtained from one specimen from Romania was also included (Table S1).

The comparison of the COI sequences showed close relatedness between mosquitoes from Germany and Italy, ranging from 100% (three samples identical to Hap2 haplotype from Italy) to a minimum identity of 97.2% (19 SNPs). Sequences from Italy and Sweden exhibited 1–16 differences (97.6–99.8% pairwise identity).

Samples from Romania clustered in two distinct groups: six specimens showed a sequence divergence of 0.3–3% with respect to the specimens from Italy, Sweden and Germany. The second group (KU214673, KU214674 and ON854129) formed a distinct cluster showing a divergence of 6–8% (Table S4).

The Iberian group (Spain and Portugal) showed a very low level of intraspecific diversity, displaying a maximum of five SNPs and 99.1–99.8% pairwise identity. The comparison between the sequences from Italy and Portugal showed the highest diversity ranging from 92.7% (48 SNPs) to 93.8% (41 SNPs).

The COI alignment (518 bp) revealed an overall pairwise identity range of 92.9–100%. In no case did the observed nucleotide variations cause stop codons, amino acid substitutions, or deletions, which excludes the presence of pseudogenes.

**Figure 2:**
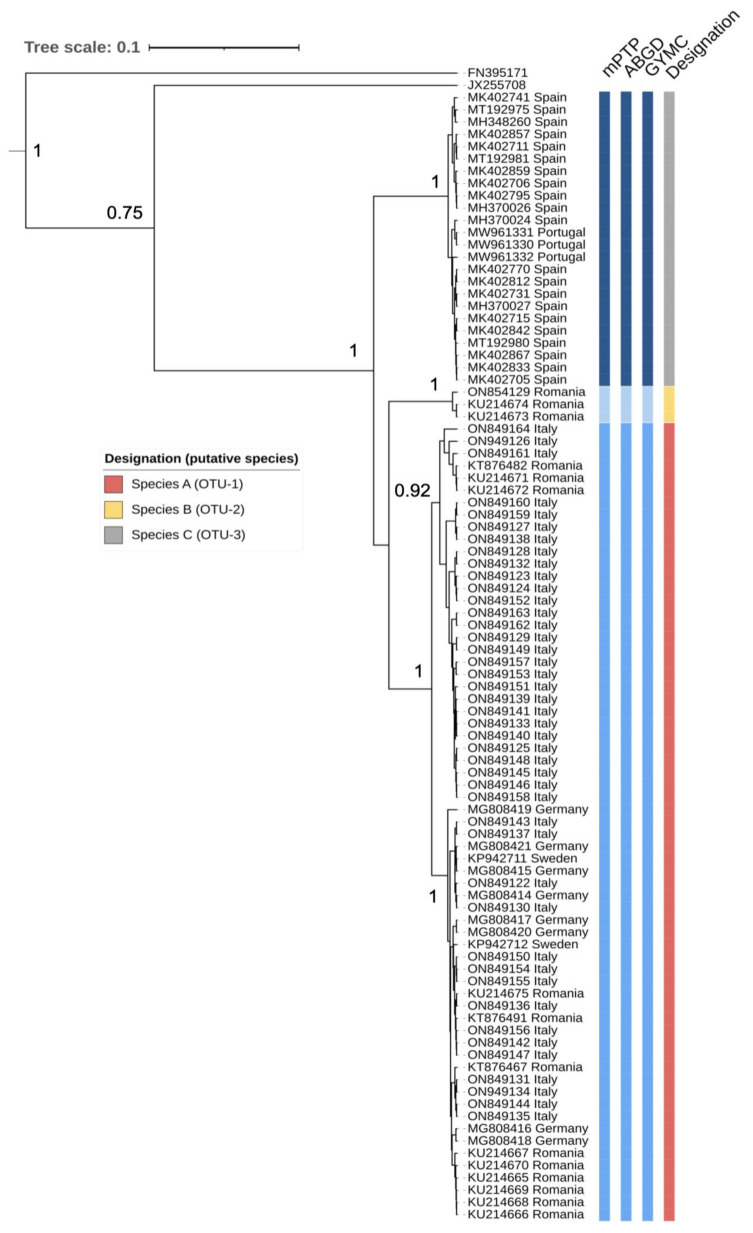
Phylogenetic and species delimitation analysis of *An. algeriensis* from Europe based on partial COI gene. Maximum clade credibility tree with numbers at branches indicating levels of support as Bayesian posterior probability >90%. *An. superpictus* (JX255708) and *Culex pipiens* biotype *molestus* (FN395171) were used as outgroup taxa. On the right are displayed the results of species delimitation analysis obtained by multi-rate Poisson tree process (mPTP), generalized mixed Yule coalescent model (GYMC), and the automatic barcode gap discoverer (ABGD). The Operational Taxonomic Units (OTUs) are also indicated.

The phylogenetic analysis showed a basal split between *An. algeriensis* from the Iberian Peninsula (Spain and Portugal) and the populations from South/Southeast/North Europe (Italy, Romania, Germany and Sweden) (Figure 2 and Figure S1). The phylogenetic split was highly supported (100% Bayesian posterior probability, BPP). The majority of haplotypes representing populations from Romania, Italy, Germany, and Sweden formed two monophyletic clades within the Operational Taxonomic Unit 1 (OTU-1) and haplotypes from Romania found at Lake Roșuleț forming OTU-2 (6.3–8% pairwise distance). The haplotypes representing OTU-3 (Spain and Portugal) showed pairwise distances of 6–7% relative to OTU-2 (Lake Roșuleț, Romania) and 6.8–8.7% when compared to the rest of the European haplotypes (OTU-1).

**Figure 3:**
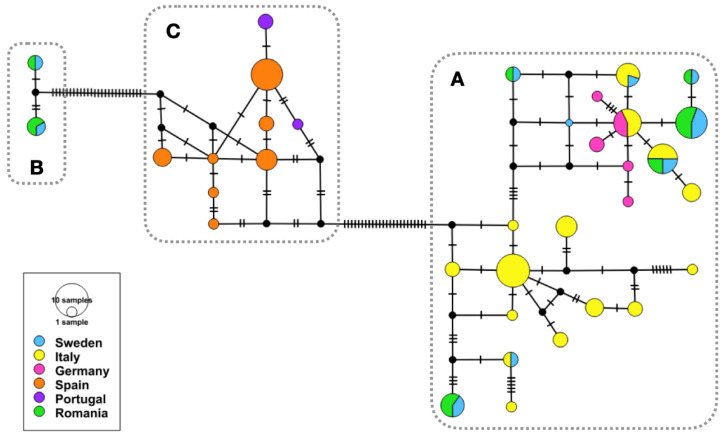
Median-joining haplotype network based on partial COI gene representing populations of *An. algeriensis* from Europe analyzed in the present study. Each coloured circle represents a haplotype. The size of the circles is proportional to haplotype frequency. Hatch marks along the connecting edges represent substitutions differentiating two haplotypes. Small black circles represent median vectors, i.e., inferred (hypothetical or unsampled) sequences required to connect existing haplotypes. The clusters marked by the grey dashed lines are named (**A**–**C**), according to the designation based on species delimitation results (see Figure 2).

All three methods employed for species delimitation yielded identical results, indicating three putative species (Species A–C) (Figure 2). This pattern was also evident when the COI dataset was used to infer a median-joining haplotype network (Figure 3).

### 2.2. Phylogenetic Analysis and Species Delimitation Based on ITS2 Sequences

A total of 83 *An. algeriensis* collected during 2020–2021 in three provinces of Southern Italy (Foggia, Taranto and Matera) were amplified and sequenced for ITS2 region (Table S1). In addition, 33 *An. algeriensis* from Klein Behnitz and Wustrow (Germany) were analyzed (Table S1). A common sequence of 545 base pairs was obtained, containing the whole ITS2 region and partial portions of the adjacent 5.8S and 28S rRNA genes (Figure S2). Through sequence analysis, no intraspecific variations were detected in sequences from Italy or Germany. Furthermore, four sequences from Sweden (Gotland island) and eight from Romania (six from Sulina and two from Roşuleţ Lake) were included in this study (Table S1). Finally, 13 sequences of this species from Spain (Navarra and La Rioja regions) were retrieved from the GenBank database (Table S2). The pairwise comparison showed 100% identity among specimens from Italy, Germany, Romania, and Sweden, whereas insertions/deletions and single nucleotide polymorphisms were observed in the sample from Spain. Specifically, all Spanish sequences showed 5 SNPs, 3 insertions (1–2 nucleotides) and 5 deletions (1–4 nucleotides). Polymorphic nucleotide positions are shown in the ITS2 alignment (Figure S2).

**Figure 4:**
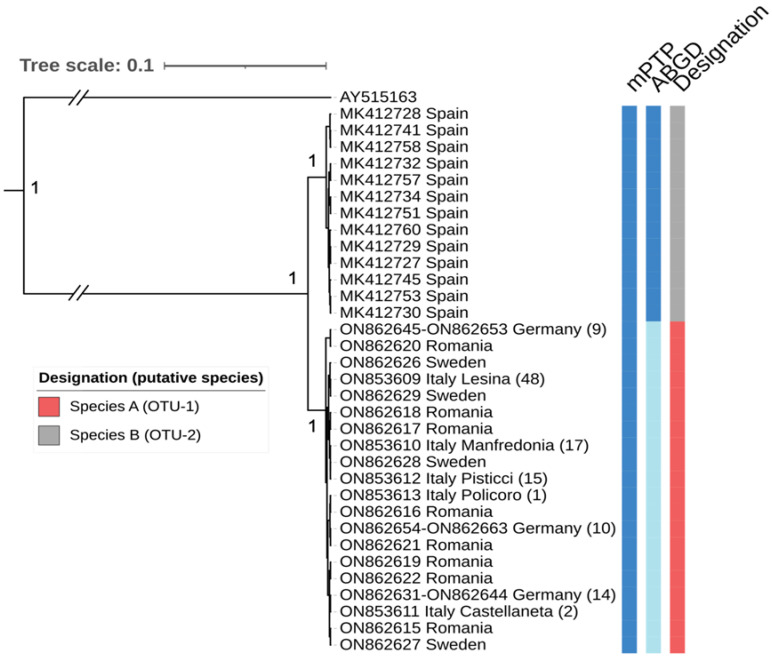
Phylogenetic and species delimitation analysis of *An. algeriensis* from Europe based on ITS2 sequences. Maximum clade credibility tree with numbers at branches indicating levels of support as Bayesian posterior probability >90%. *An. superpictus* (AY515163) was used as outgroup taxa. On the right are displayed the results of species delimitation analysis obtained by multi-rate Poisson tree process (mPTP) and the automatic barcode gap discoverer (ABGD). The Operational Taxonomic Units (OTUs) are also indicated.

ITS2 phylogenetic analysis showed the same major split between populations from the Iberian Peninsula (Spain) and those from Italy, Romania, Germany, and Sweden (Figure 4), with high BPP support (100%). The two clades showed a minimum interspecific pairwise distance of 2.3%. The low sequence heterogeneity of the ITS2 dataset precluded the use of GYMC. The other two algorithms, one based on fitting branching events to an exponential distribution for each species (mPTP) and the other based on the discovery of a barcoding gap (ABGD) returned different delimitation results. The mPTP partition placed the two phylogenetic clades in a single group, whereas the ABGD separated the Iberian clade (Spain, Species B) from the rest of the European populations (Species A).

## 3. Discussion

A recent COI-based study of mosquitoes from Spain found evidence of cryptic speciation of *An. algeriensis*, indicating two morphologically indistinguishable cryptic species [25]. Similar to our results, the authors found a high level of divergence (~5%) between mosquitoes from Spain and those from Germany and Sweden. In our analysis of COI sequences, all three species delimitation methods indicated the existence of three putative species (Species A–C) represented by well-supported phylogenetic clades and high genetic divergence (minimum interspecific distance between Species B–C = 5.8%, Species A–C = 6%, Species A–B = 5.8%). Although we found a discordance in phylogenetic topology and delimitation methods between nDNA and mtDNA markers, all analyses indicate a minimum of two putative species, confirming the observation of Delgado-Serra et al. [25] that *An. algerienis* in Europe undergoes cryptic speciation. An interesting case is that of Species B (out-2) observed by COI analysis and represented by specimens collected in Danube Delta at Lake Roșuleț (Romania). This species showed high genetic divergence relative to mosquitoes collected ~10km north (Sulina, Danube Delta), which are conspecifics of Species A members from Italy, Germany, and Sweden. The number of specimens from Species B is low (n = 3) in our study, which precludes a comprehensive analysis of its genetic variation. However, it would be worthwhile to test whether the highly heterogeneous landscape of the Danube Delta ecosystem complex [10] is contributing to speciation in *An. algeriensis* at such a small geographic scale.

Despite incongruent tree topologies, the results confirm a split of European *An. algeriensis* populations, where the Iberian populations have accrued observable change in nDNA. The major phylogenetic clades (Iberian and rest of Europe) are well-supported, and in both cases the former showed the highest divergence, suggesting that the mosquitoes from Spain and Portugal may represent older populations than those of the rest of Europe.

Although ITS2 has been shown to be an effective marker for anopheline mosquitoes [29], in the case of *An. algeriensis* it may be that the populations from Southern, Southeastern, and Northern Europe have been able to mix relatively recently while still containing cases indicating isolation (Species B in Danube Delta). Phylogeographic patterns of this taxon could be clarified by more extensive sampling and by the use of additional markers [30]. It would also be advisable to carefully review the diagnostic characters of *An. algeriensis*, verifying whether there are morphological differences across the taxon’s geographical range.

In conclusion, the present analysis supports the hypothesis that *An. algeriensis* is a species complex comprising a minimum of two (probably three) different cryptic species.

## 4. Materials and Methods

### 4.1. Mosquito Collection and Processing

In July and September 2020, *Anopheles algeriensis* specimens were collected within the framework of an entomological survey conducted in in the Gargano promontory (Foggia Province) in Apulia [20]. In September 2021, a further survey was carried out on several horse farms in Castellaneta (Taranto Province), in Apulia, and in Pisticci and Policoro (Matera Province), in Basilicata (Figure 1). Mosquitoes were collected by different methods, as described by Raele et al. [20]. Adult mosquitoes were identified according to morphological keys [31] and stored at −20 °C for molecular analysis. Single mosquito legs or extracted DNA from 33 *An. algeriensis* specimens (Table S1) collected in 2015–2017 in two Northeast German localities (Klein Behnitz and Wustrow) [13] were available for comparative analyses.

### 4.2. Polymerase Chain Reaction (PCR) and Sequencing

A fragment of the COI gene from the *An. algeriensis* caught in Italy and one specimen from Romania were Sanger-sequenced using primers described by Folmer et al. [32]. For mosquitoes from Italy and Germany, the nuclear ribosomal ITS2 region, including partial sequences of the 18S and 5.8S rRNA genes, was amplified using primers designed by Marinucci et al. [26]. For both molecular barcodes, PCR reactions were performed using one mosquito leg directly as a template for amplification or approximately 25–50 nanograms of DNA extracted from each individual mosquito by means of a PureLink Genomic DNA Kit (Thermo Fisher Scientific). All reactions were performed in a final volume of 25 μL with the following reagent concentrations: 0.1 units/µL of EconoTaq DNA polymerase, 1X reaction buffer (pH 9.0), 400 µM of each dNTP, 3 mM MgCl2 (EconoTaq PLUS GREEN, Lucigen), and 10 picomoles of each primers. For sequencing, ~25 ng of purified PCR product (estimated by comparison with standards on stained agarose gels) was mixed with 25 picomoles of each forward and reverse primers and directly sequenced at Eurofins Genomics (Ebersberg, Germany).

In addition to ITS2 sequences generated as described above, original ITS2 sequences from *An. algeriensis* specimens from Sweden and Romania, directly provided by some authors, were made available for the comparative analyses. All COI and ITS2 sequences obtained in this study were submitted to GenBank (Table S1).

### 4.3. Data Analysis

Multiple sequence alignment barcodes were generated for each molecular barcode with the MAFFT algorithm implemented in Geneious Prime (Biomatters). Sequences were trimmed to identical lengths: 518 base pairs for COI and 503 base pairs for the ITS2 marker region.

We used maximum likelihood (ML) and Bayesian inference (BI) to analyze the phylogenetic relationship between different European populations of *An. algeriensis*. Using MEGA11 [33], we selected the best-fitting nucleotide substitution model based on the lowest Bayesian information criterion (BIC) and constructed ML trees for the two markers, with branch support estimated by 1000 bootstrap replicates (Figure S1). Bayesian inference was conducted by Markov chain Monte Carlo sampling in BEAST v1.10.4 [34], where one run of 10 million generations was sampled every 5000 iterations. Convergence was assessed with Tracer 1.7 [35] and the final tree topology was summarized with TreeAnnotator 1.10 after a burn-in of 10%. Resulting BI phylogenies were edited and annotated in iTOL [36].

We employed three algorithms for species delimitation, namely the multi-rate Poisson tree processes (mPTP) [37], the generalized mixed Yule coalescent model (GYMC) [38,39], and the automatic barcode gap discoverer (ABGD) [40] (Supplementary File S1). Distance matrices were calculated with Mega11, applying the Kimura 2-parameter model [41] (Table S4) to determine the minimum intraspecific and maximum interspecific genetic distances. Operational taxonomic units (OTUs) were defined. Finally, to visualize the levels of intraspecific and interspecific differentiation, we constructed median joining haplotype networks in PopART 1.7 [42].

## Data Availability

Not applicable.

## Supplementary Materials

The following supporting information can be downloaded at: https://www.mdpi.com/article/10.3390/pathogens11090990/s1. Table S1: List of *Anopheles algeriensis* specimens analyzed in the present study; Table S2: List of additional COI and ITS2 sequences, retrieved from GenBank, used as comparison in the present study; Table S3: List of COI haplotypes identified in *An. algeriensis* specimens in the present study; Table S4: Distance matrices from aligned COI nucleotide sequences by using the Kimura 2-parameter model; Figure S1: Maximum likelihood phylogenetic tree of partial COI gene sequences representing European populations of *Anopheles algeriensis*; Figure S2: Sequence alignment of the ITS2 of *Anopheles algeriensis*; and File S1: Algorithms used for species delimitation analysis.
