# Supplementary material for: Molecular Characterization of Anopheles algeriensis Theobald, 1903 (Diptera: Culicidae) Populations from Europe
Source: Pathogens. 2022 Aug 30;11(9):990. doi: 10.3390/pathogens11090990 (PMC9505642; doi:10.3390/pathogens11090990)

Figure S1. Cluster analysis of partial COI gene sequences, representing European populations of *Anopheles algeriensis,* based on Maximun Likelihood method. The tree was built using the T92+G model and rooted with *An. superpictus* and *Culex pipiens molestus*. Statistical support of taxa clustering was assessed by 1000 bootstrap iterations and support values ≥70% are displayed at tree nodes. Tree scale indicates the amount of genetic divergence in substitution per site.*Original sequences obtained in the present study.

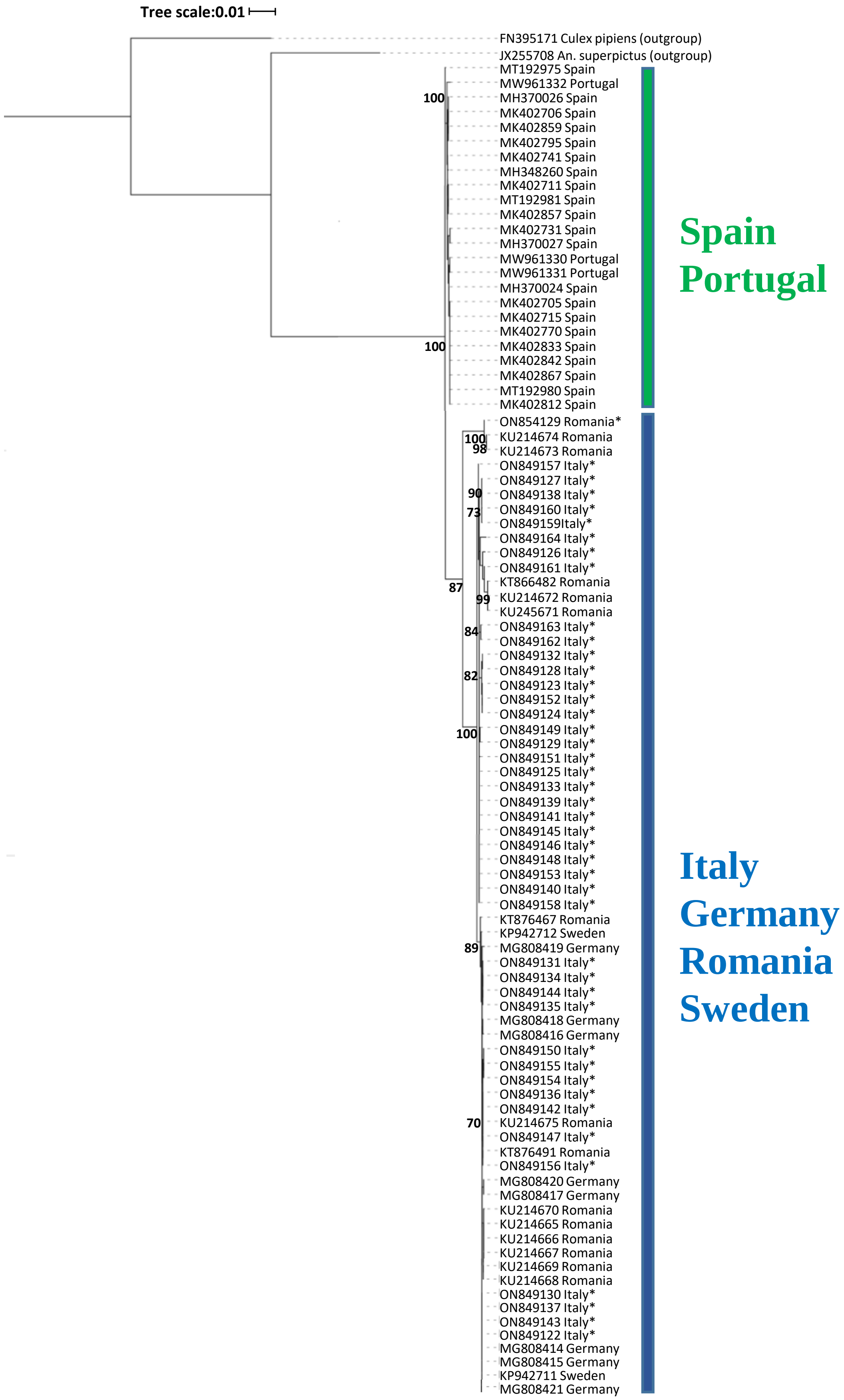

Supplement: Supplementary file 1 [file pathogens-11-00990-s001.zip › Figure S1.pdf]
