# Supplementary material for: Molecular Characterization of Anopheles algeriensis Theobald, 1903 (Diptera: Culicidae) Populations from Europe
Source: Pathogens. 2022 Aug 30;11(9):990. doi: 10.3390/pathogens11090990 (PMC9505642; doi:10.3390/pathogens11090990)

Figure S2. Sequence alignment of the ITS2 of *Anopheles algeriensis*. The five sequences shown in the alignment are representative of the *Anopheles algeriensis* samples examined for each geographic area. The colored nucleotides indicate the position of single nucleotide polymorphisms. Red asterisks positioned in correspondence with the dash (-) highlight the position of insertions or deletions in the alignment. Start end of the presumptive ITS2 region are indicated by red arrows.

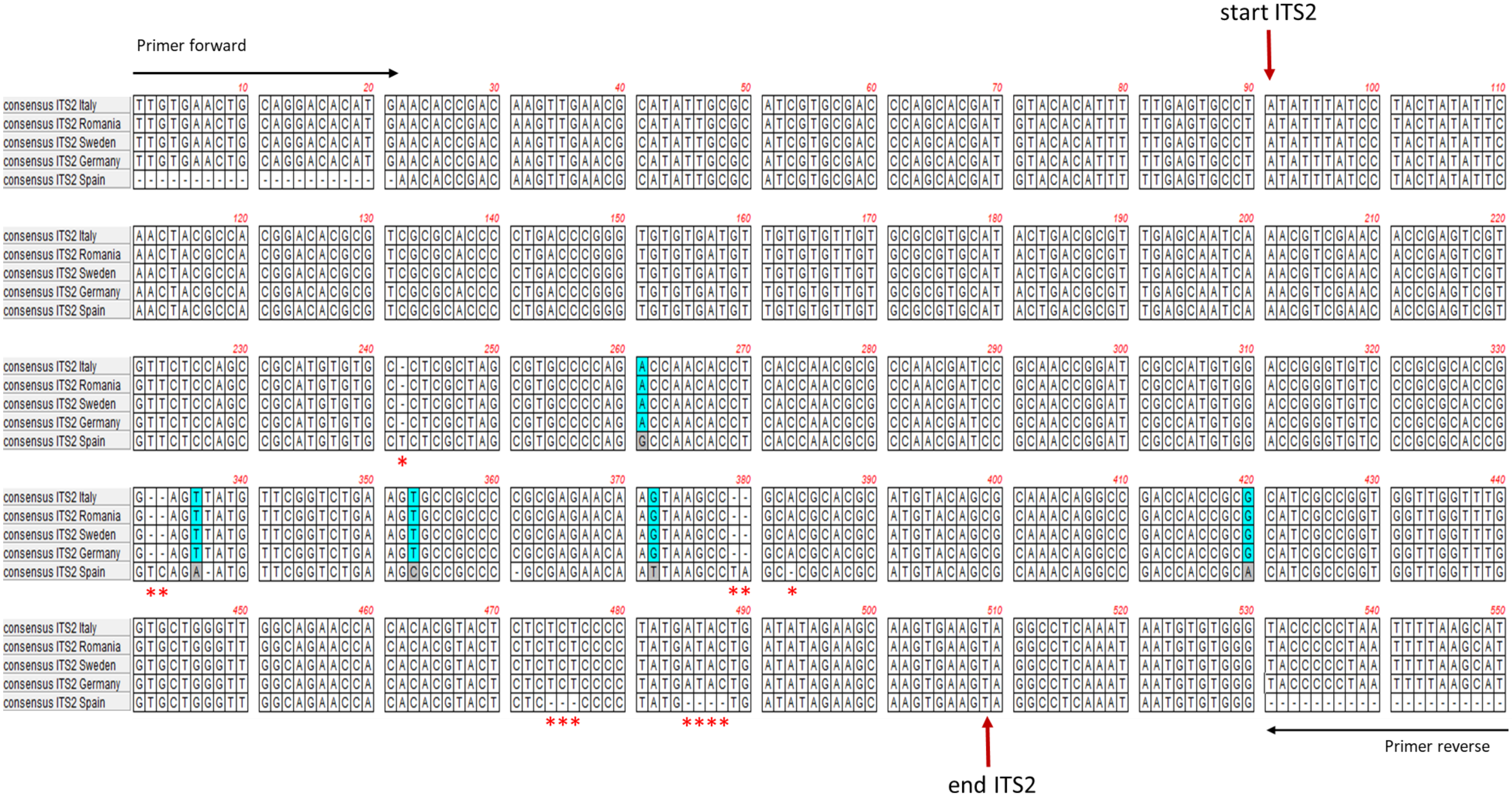

Supplement: Supplementary file 1 [file pathogens-11-00990-s001.zip › Figure S2.pdf]
