# Supplementary material for: Molecular Characterization of Anopheles algeriensis Theobald, 1903 (Diptera: Culicidae) Populations from Europe
Source: Pathogens. 2022 Aug 30;11(9):990. doi: 10.3390/pathogens11090990 (PMC9505642; doi:10.3390/pathogens11090990)
Supplement: Supplementary file 1 [file pathogens-11-00990-s001.zip › File S1.pdf]

Supplementary File S1: Information on the three algorithms used for species delimitation.

ABGD

COI_distances:
Pmin=0.001
Pmax=0.1
Steps=10
X=3
distance=K80, TS/TV=2.0
Nb bins=39

COI_results:
Nb seq:92 */
Partition 1 : found 23 groups (prior maximal distance P= 0.001000)
Partition 2 : found 23 groups (prior maximal distance P= 0.001668)
Partition 3 : found 8 groups (prior maximal distance P= 0.002783)
Partition 4 : found 3 groups (prior maximal distance P= 0.004642)
Partition 5 : found 3 groups (prior maximal distance P= 0.007743)
Partition 6 : found 3 groups (prior maximal distance P= 0.012915)

ITS2_distances:
Pmin=0.001
Pmax=0.1
Steps=7
X=2
distance=K80, TS/TV=2.0
Nb bins=10

ITS2_results:
Nb seq:33 */
Partition 1 : found 2 groups (prior maximal distance P= 0.001000)
Partition 2 : found 2 groups (prior maximal distance P= 0.002154)
Partition 3 : found 2 groups (prior maximal distance P= 0.004642)
Partition 4 : found 2 groups (prior maximal distance P= 0.010000)
Partition 5 : found 2 groups (prior maximal distance P= 0.021544)

GYMC

method: single
likelihood of null model: 810.3946
maximum likelihood of GMYC model: 815.6901
likelihood ratio: 10.59099
result of LR test: 0.005014135**

number of ML clusters: 3
confidence interval: 1-15

number of ML entities: 5
confidence interval: 3-22

threshold time: -0.0167607

mPTP

COI results:

Command: ../bin/mptp mptp --ml --multi --outgroup FN395171,JX255708 --outgroup_crop --svg_fontsize 13 --tree_file ../uploads/hhib50q6ikaju10b9oc9hk2h22.newick --output_file ../uploads/hhib50q6ikaju10b9oc9hk2h22.1
Number of edges greater than minimum branch length: 58 / 182
Null-model score: 269.946667
Best score for multi coalescent rate: 269.946667
Number of delimited species: 3

Species 1:
KU214669_Romania
KU214670_Romania
KU214668_Romania
KU214667_Romania
KU214666_Romania
KU214665_Romania
ON849143_Italy
ON849137_Italy
ON849130_Italy
ON849122_Italy
MG808421_Germany
MG808415_Germany
MG808414_Germany
KP942711_Sweden
MG808417_Germany
MG808420_Germany
MG808416_Germany
MG808418_Germany
ON849154_Italy
ON849155_Italy
ON849150_Italy
KT876491_Romania
KU214675_Romania
ON849136_Italy
ON849142_Italy
ON849147_Italy
ON849156_Italy
ON849131_Italy
ON849135_Italy
ON849144_Italy
ON949134_Italy
MG808419_Germany
KP942712_Sweden
KT876467_Romania
ON849157_Italy
ON849159_Italy
ON849160_Italy
ON849138_Italy
ON849127_Italy
KU214671_Romania
KU214672_Romania
KT876482_Romania
ON849161_Italy

ON949126_Italy
ON849164_Italy
ON849124_Italy
ON849152_Italy
ON849123_Italy
ON849128_Italy
ON849132_Italy
ON849162_Italy
ON849163_Italy
ON849129_Italy
ON849149_Italy
ON849125_Italy
ON849133_Italy
ON849139_Italy
ON849140_Italy
ON849141_Italy
ON849145_Italy
ON849146_Italy
ON849148_Italy
ON849153_Italy
ON849158_Italy
ON849151_Italy
Species 2:
ON854129_Romania
KU214673_Romania
KU214674_Romania
Species 3:
MW961332_Portugal
MT192975_Spain
MH370026_Spain
MK402706_Spain
MK402795_Spain
MK402859_Spain
MH370027_Spain
MK402731_Spain
MK402857_Spain
MT192981_Spain
MK402711_Spain
MK402741_Spain
MH348260_Spain
MH370024_Spain
MK402705_Spain
MK402715_Spain
MK402770_Spain
MK402812_Spain
MK402833_Spain
MK402842_Spain
MK402867_Spain
MT192980_Spain
MW961330_Portugal
MW961331_Portugal

ITS2 results:
Command: ../bin/mptp mptp --ml --multi --outgroup AY515163 --outgroup_crop --tree_file ../uploads/h10tkd822rn5jj767npvoi9um0.newick --output_file ../uploads/h10tkd822rn5jj767npvoi9um0.1
Number of edges greater than minimum branch length: 2 / 64
Null-model score: 6.950537
Best score for multi coalescent rate: 6.950537
Number of delimited species: 1

Species 1:
ON862628_Sweden
ON862628_Sweden
ON862627_Sweden
ON862626_Sweden
ON862622_Romania
ON862621_Romania
ON862620_Romania
ON862619_Romania
ON862618_Romania
ON862617_Romania
ON862616_Romania
ON862615_Romania
ON853613 Italy Policoro (1)
ON853612 Italy Pisticci (15)
ON853610 Italy Menfredonia (17)
ON853609 Italy Lesina (48)
ON862645-ON862653 Germany (9)
ON862654-ON862663 Germany (10)
ON862631-ON862644 Germany (14)
ON853611 Italy Castellaneta (2)
MK412727_Spain
MK412728_Spain
MK412729_Spain
MK412730_Spain
MK412732_Spain
MK412734_Spain
MK412741_Spain
MK412745_Spain
MK412751_Spain
MK412753_Spain
MK412757_Spain
MK412758_Spain
MK412760_Spain
