# Supplementary material for: Molecular Characterization of Anopheles algeriensis Theobald, 1903 (Diptera: Culicidae) Populations from Europe
Source: Pathogens. 2022 Aug 30;11(9):990. doi: 10.3390/pathogens11090990 (PMC9505642; doi:10.3390/pathogens11090990)
Supplement: Supplementary file 1 [file pathogens-11-00990-s001.zip › Table S1.pdf]

Table S1. List of *Anopheles algeriensis* specimens analyzed in the present study.

Country	Collection site	Collection Year	No. Specimens analysed for ITS2	GenBank ITS2 Acc. Num.	No. Specimens analysed for COI	GenBank COI Acc. Num.
Italy (No. Tot 83)	Lesina (Foggia), Apulia	2020	48	ON853609*	39	ON849122-ON849160
	Manfredonia (Foggia), Apulia	2020	17	ON853610*	1	ON849161
	Castellaneta (Taranto, Apulia	2021	2	ON853611*	2	ON849163-ON849164
	Pisticci (Matera). Basilicata	2021	15	ON853612*	1	ON849162
	Policoro (Matera), Basilicata	2021	1	ON853613*	---	---
Germany (No. Tot 33)	Klein Behnitz, Brandenburg	2016	14	ON862631-ON862644	---	---
	Wustrow, Mecklenburg-Western Pomerania	2017	9	ON862645-ON862653	---	---
	Wustrow, Mecklenburg-Western Pomerania	2015	10	ON862654-ON862663	---	---
Romania (No. Tot 8)	Sulina, Danube Delta Biosphere Reserve	2014	5	ON862615-ON862619	---	---
	Sulina, Danube Delta Biosphere Reserve	2015	1	ON862620	---	---
	Lake Rosulet, Danube Delta Bioshere Reserve	2015	1	ON862621	1	ON854129
	Lake Rosulet, Danube Delta Bioshere Reserve	2016	1	ON862622	---	---
Sweden	Gotland	2013	4	ON862626-ON862629	---	---

* One representative ITS2 sequence for each Italian collection site was submitted in GenBank.
