# Supplementary material for: Molecular Characterization of Anopheles algeriensis Theobald, 1903 (Diptera: Culicidae) Populations from Europe
Source: Pathogens. 2022 Aug 30;11(9):990. doi: 10.3390/pathogens11090990 (PMC9505642; doi:10.3390/pathogens11090990)
Supplement: Supplementary file 1 [file pathogens-11-00990-s001.zip › Table S2.pdf]

Table S2. List of additional COI and ITS2 sequences, retrieved from GenBank, used as comparison in the present study.

**Country**	**Locality**	**No. specimens**	**COI Genbank Accession No.**
Germany	Klein Behnitz; Wustrow	8	MG808414 to MG808421
Portugal	Santiago do Cacém	3	MW961330 to MW961332
Romania	Danube Delta	14	KU214665 to KU214675<br>KT876467<br>KT876482<br>KT876491
Spain	Navarra; La Roja; Maiorca	21	MK402705-MK402706<br>MK402711<br>MK402715<br>MK402731<br>MK402741<br>MK402770<br>MK402795<br>MK402812<br>MK402833<br>MK402842<br>MK402857<br>MK402859<br>MK402867<br>MH370024<br>MH370026-MH370027<br>MT192975<br>MT192980-MT192981<br>MH348260
Sweden	Gotland	2	KP942711-KP942712
**Country**		**No. specimens**	**ITS2 GenBank Accession No.**
Spain	Navarra; La Roja	13	MK412727 to MK412730<br>MK412732<br>MK412734<br>MK412741<br>MK412745<br>MK412751<br>MK412753<br>MK412757-MK412758<br>MK412760
**Outgroup**		**Species**	**GenBank Accession No.**
ITS2		*Anopheles superpictus*	AY515163
COI		*Anopheles superpictus*<br>*Culex pipiens*	JX255708<br>FN395171
